# Integrated Analyses of the Mechanism of Flower Color Formation in Alfalfa (*Medicago sativa*)

**DOI:** 10.3390/metabo15020135

**Published:** 2025-02-17

**Authors:** Zhaozhu Wen, Huancheng Liu, Qian Zhang, Xuran Lu, Kai Jiang, Qinyan Bao, Zhifei Zhang, Guofeng Yang, Zeng-Yu Wang

**Affiliations:** 1 College of Agronomy, Hunan Agricultural University, Changsha 410128, Chinazhangzf@hunau.edu.cn (Z.Z.); 2 College of Grassland Science, Qingdao Agricultural University, Qingdao 266109, China

**Keywords:** alfalfa, *Medicago sativa*, flower color, anthocyanins, carotenoids, delphinidin-3,5-*O*-diglucoside

## Abstract

Background: Alfalfa (*Medicago sativa*) is one of the most valuable forages in the world. As an outcrossing species, it needs bright flowers to attract pollinators to deal with self-incompatibility. Although various flower colors have been observed and described in alfalfa a long time ago, the biochemical and molecular mechanism of its color formation is still unclear. Methods: By analyzing alfalfa lines with five contrasting flower colors including white (cream-colored), yellow, lavender (purple), dark purple and dark blue, various kinds and levels of anthocyanins, carotenoids and other flavonoids were detected in different colored petals, and their roles in color formation were revealed. Results: Notably, the content of delphinidin-3,5-*O*-diglucoside in lines 3, 4 and 5 was 58.88, 100.80 and 94.07 times that of line 1, respectively. Delphinidin-3,5-*O*-diglucoside was the key factor for purple and blue color formation. Lutein and β-carotene were the main factors for the yellow color formation. By analyzing differentially expressed genes responsible for specific biochemical pathways and compounds, 27 genes were found to be associated with purple and blue color formation, and 14 genes were found to play an important role in yellow color formation. Conclusions: The difference in petal color between white, purple and blue petals was mainly caused by the accumulation of delphinidin-3,5-O-diglucoside. The difference in petal color between white and yellow petals was mainly affected by the production of lutein and β-carotene. These findings provide a basis for understanding the biochemical and molecular mechanism of alfalfa flower color formation.

## 1. Introduction

Flower color is an important trait for plants as visual signaling to attract pollinators and ensure their reproductive success [1,2]. Some floral species, such as orchids, are characterized by their fantastic floral morphology and high levels of corolla color polymorphism and variability, which have played an important role in pollination [1,3]. As one of the most important forages in the world, alfalfa (*Medicago sativa* L., 2n = 4x = 32) is a typical cross-pollinated plant with a high degree of self-incompatibility, attracting insect pollinators using its bright flowers [4,5,6].

Flavonoids and carotenoids are the two major floral pigments, and anthocyanins constitute the largest group of flavonoids. The biosynthesis pathway of anthocyanins, which affect plant flower colors, is regulated by many genes. *4CL* (*4-coumarate: CoA ligase*), *CHS* (*chalcone synthase*), *CHI* (*chalcone isomerase*), *F3′H* (*flavonoid 3′-hydroxylase*), *F3′5′H* (*flavonoid 3′,5′-hydroxylase*) and *DFR* (*dihydroflavonol reductase*) are important enzymatic genes in the upstream anthocyanin synthesis pathway. Among them, *4CL* not only participates in the biosynthesis of anthocyanin but also is involved in lignin formation and the production of other phenolic compounds [7]. *F3′H* and *F3′5′H* can change the type of flavonoids, transforming naringenin/dihydrokaempferol into eriodictyol/dihydroquercetin and dihydrotricetin/dihydromyricetin [8]. Blocking the synthesis of *F3′5′H* affects the accumulation and reduction in anthocyanins, leading to a light flower color in pea (*Pisum sativum*) [9]. Arabidopsis *DFR* mutant prevents dihydroplavonones from being reduced to leucopelargonidin, making the seed coat white, similar to the *f3′h* mutant phenotype [10,11]. At the downstream of the anthocyanin biosynthesis pathway, anthocyanidins can form anthocyanins through glycosylation, methylation, and acylation reactions under the action of various UDP-glycosyltransferases (*UGTs*). This process makes anthocyanins more stable in property, more abundant in color, and more complex in structure.

Genes in the downstream of anthocyanin biosynthesis are regulated by the ternary complexes of transcription factors, named MYB-bHLH-WD40 (MBW). These MBW complexes include MYB (such as PAP1 and TT2), bHLH (such as TT8, GL3, and EGL3), and WD40 proteins (such as TTG1) [12,13,14,15]. The conservatism of the interaction of MYB, bHLH, and WD40 in controlling proanthocyanidins (PAs) and anthocyanin biosynthesis has been verified in many plants [16]. MBW usually targets the promoters of structural genes to activate the transcription of these genes in plants [12]. MtTT8 interacts with MYB and MtWD40-1 to form a regulatory complex that regulates the anthocyanin biosynthesis genes and affects the seed coat and flower color in *Medicago truncatula* [17]. Allelic variations in the promoters between *GhTT19*LW and *GhTT19*LR, rather than genic regions, are the genetic cause of petal color variations in cotton [18].

Flavonoids, especially anthocyanins, help to produce a variety of flower colors ranging from light yellow to orange, red, blue, purple, and dark purple, whereas carotenoids provide yellow, orange, and bright red colors to flowers. With C6-C3-C6 as the carbon skeleton, anthocyanins form the basic structures of delphinidin, peonidin, petudinin, malvidin, pelargonidin, and cyanidin through different combinations, which react with different sugar, methyl, and acyl groups to produce more than 600 anthocyanins [19]. The main forms of anthocyanins vary among plant species and in different organs. Cyanidins and pelargonidins are the main pigments in red plant organs, while delphinidins, petudinins, and malvidins are important chromogenic substances in many blue/purple plant organs [20].

The biosynthesis of carotenoids also is a complex process involving multiple enzymatic reactions; PSY (*phytoene synthase*) is located upstream of carotenoid biosynthesis, while ZEP (*zeaxanthin epoxidase*) is located downstream [21]. In most plants, LcyB (lycopene beta cyclase) and LcyE (lycopene epsilon cyclase) both use lycopene as a substrate to synthesize δ-carotene and λ-carotene, respectively, altering the composition of carotenoids [22].

To understand the key metabolic compounds and the associated gene expression networks controlling flower color formation in alfalfa, multiple lines with contrasting flower colors were analyzed by metabolic profiling and RNA-seq. Combined analysis revealed specific compounds and genes affecting flower color formation in alfalfa. This study provides new insights into the biochemical and molecular mechanisms associated with the biosynthesis and regulation of anthocyanins and carotenoids in alfalfa flowers.

## 2. Materials and Methods

### 2.1. Plant Materials and Growth Conditions

Alfalfa lines of the “Wudi” population used in this study were grown in Wudi County (117.97, 37.92), Jiaozhou Experimental Station (120.10, 36.31), and Qingdao Agricultural University (120.40, 36.32), Shandong Province, China. Petals were collected from fully opened petals, and 3.0 g of the collected petals per sample were used for metabolic analysis and RNA-seq analysis.

### 2.2. Scanning Electron Microscopy (SEM) Analysis

For the HITACHI Regulus 8100 scanning electron microscopy examination, fully opened petals were collected in different color alfalfa lines. Samples were fixed, dried, and sputter-coated as reported previously [23].

### 2.3. Metabolic Analysis

The petal samples were freeze-dried and ground into powder (30 Hz, 1.5 min). Then, 50 mg of the powdered sample was weighed, used for extraction with 0.5 mL methanol/water/hydrochloric acid (500:500:1, *v*/*v*/*v*), and vortexed and ultrasonicated for 5 min. The samples were then centrifuged at 12,000× *g* for 3 min at 4 °C. The supernatant of each sample was filtered through a membrane filter (0.22 μm, Anpel, Shanghai, China).

The sample extracts of anthocyanins and flavonoids were analyzed using a UPLC-ESI-MS/MS system (UPLC, ExionLC™ AD, https://sciex.com.cn/ (accessed on 15 August 2022); MS, Applied Biosystems 6500 Triple Quadrupole, ESI Turbo Ion-Spray interface). And carotenoids were analyzed using a UPLC-APCI-MS/MS system (APCI Heated Nebulizer). The anthocyanin, flavonoid, and carotenoid contents were detected using MetWare (http://www.metware.cn/ (accessed on 6 December 2022)) based on the AB Sciex QTRAP 6500 LC-MS/MS platform.

### 2.4. RNA-Seq Analysis

Total RNA was extracted from the alfalfa petals using the TRIzol^®^ Reagent Kit (Invitrogen, CA, USA). To evaluate the quality of the extracted RNA, the 2100 Bioanalyzer (Agilent, CA, USA) was utilized, and the ND-2000 (NanoDrop Technologies, DE, USA) was used for quantification. RNA-seq transcriptome libraries were sequenced using the Illumina HiSeq xten/NovaSeq 6000 sequencer (Illumina, San Diego, CA, USA). For the synthesis of double-stranded cDNA, a SuperScript double-stranded cDNA synthesis kit (Invitrogen, CA, USA) was used in combination with random hexamer primers (Illumina). After the quantification of the product using TBS380, sequencing was performed on the Illumina HiSeq xten/NovaSeq 6000 sequencer.

The clean reads were mapped and annotated following the reference genome with orientation mode using the HISAT2 software [4,24]. Essentially, differential expression analysis was performed using the DEGseq with Q value ≤ 0.05, and DEGs with |log2FC| > 1 and Q value ≤ 0.001 were significant DEGs. KEGG functional enrichment and pathway analyses were carried out using Goatools and KOBAS [25].

### 2.5. Statistical Analysis

Cluster analysis and variance analysis were performed using SPSS v.0. Principal component analysis was performed using MetWare 2.0.

## 3. Results

### 3.1. Characterization of Alfalfa Lines with Different Flower Colors

**Figure 1:**
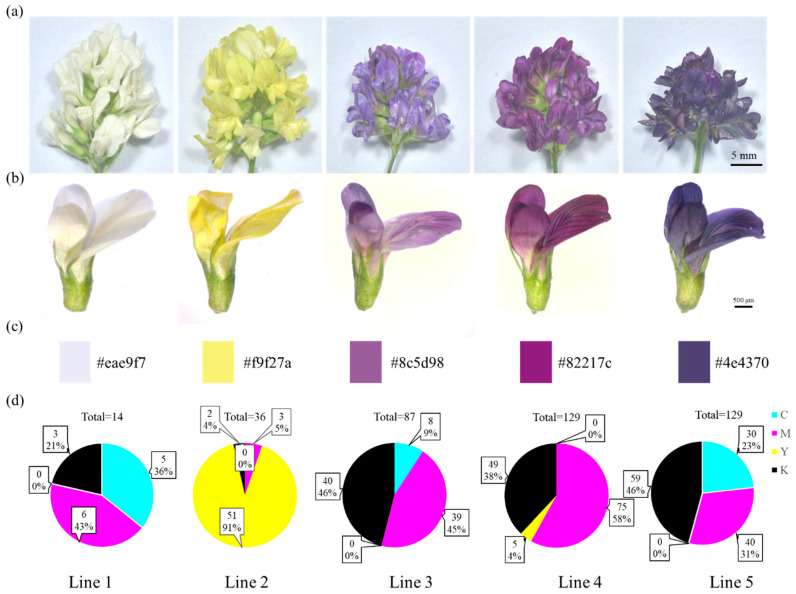
Characterization of flower colors in different alfalfa lines. (**a**) Inflorescence color of different alfalfa lines. (**b**) Flower color of different alfalfa lines. (**c**) Red, green, and blue (RGB) color chart of alfalfa petals. (**d**) Cyan, magenta, yellow, and black (CMYK) color chart of alfalfa petals.

In contrast to the common “lavender” flowers in alfalfa, the “Wudi” population has amazing color polymorphism, including white (cream-colored), yellow, lavender (purple), dark purple, dark blue, and some intermediate colors. Growing the materials in distant locations did not affect the flower color, and the coloration remained consistent throughout the perennial life cycle of the plants. Thus, variation in floral color is likely genetically determined rather than a result of, for example, soil chemistry. Lines 1 (white/cream-colored), 2 (yellow), 3 (lavender/purple), 4 (dark purple), and 5 (dark blue) with obvious color differences were selected from the “Wudi” population (Figure 1a,b). The colors of the style, stigma, ovary, filament, and anther were indistinguishable after removing the petals (Supplementary Figure S1). Therefore, the pigment difference in alfalfa flowers lies on the petal rather than on other organs.

To identify the petal color more clearly, the petal colors of lines 1–5 were measured with getcolor. The petal colors of fully opened flowers were calibrated on the red, green, and blue (RGB) scale, and the specific RGB values of petals 1–5 were determined as #eae9f7, #f9f27a, #8c5d98, #82217c, and #4e4370, respectively (Figure 1c). According to the cyan, magenta, yellow, and black (CMYK) color scale (line 5), it is 30.4% cyan, 40.2% magenta, 0% yellow, and 56.1% black (Figure 1d). This result suggests that the lavender, dark purple, and dark blue petal colors of lines 3–5 are caused by the addition of different degrees of purple obtained from the blending of magenta and black to the white base of line 1 (Figure 1c,d).

Cell morphology of the deepest colored regions of the petals was observed by scanning electron microscopes to determine the influence of epidermal cells on petal color. No significant difference was found in petal epidermal cell structures between the five lines (Supplementary Figure S2).

### 3.2. Anthocyanin Content Is Much Higher in Purple and Blue Petals

Metabolic analysis of flavonoids, anthocyanins, and carotenoids was conducted using petals collected from different colored alfalfa lines.

**Figure 2:**
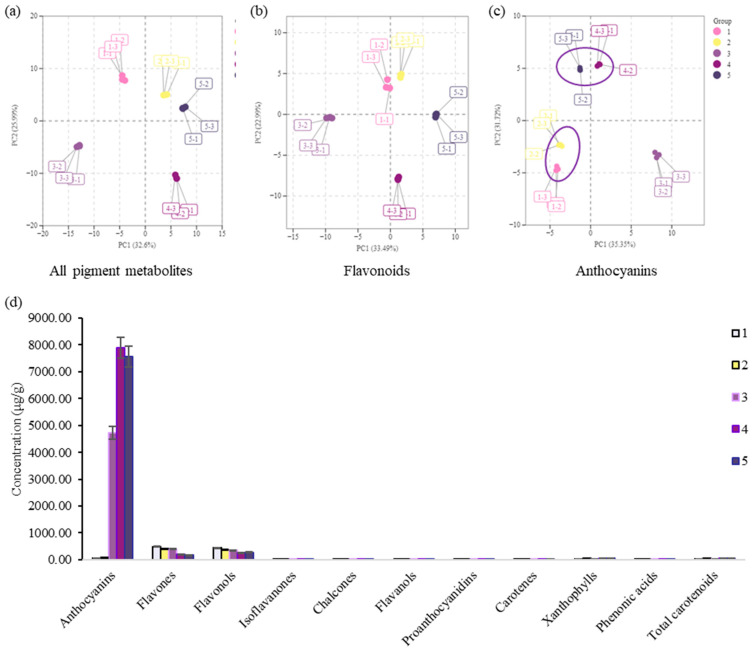
Principal component analysis (PCA) and pigment content in alfalfa lines 1–5. (**a**) PCA of all pigment metabolites. (**b**) PCA of flavonoids (excluding anthocyanins). (**c**) PCA of anthocyanins. (**d**) Pigment content of alfalfa lines (1–5) with different petal colors. Error bars represent standard deviations (SDs) of three biological replicates.

Principal component analysis (PCA) of all pigment metabolites and flavonoids did not place the samples into groups consistent with petal colors (Figure 2a,b). However, PCA of anthocyanins placed the samples into three groups consistent with petal colors. Lines 1 and 2 were in the light color group, and line 3 was in the normal color group, while lines 4 and 5 were in the dark color group, suggesting that anthocyanins play a critical role in purple and blue color determination (Figure 2c).

The contents of different pigments were analyzed in the petals. The results showed that anthocyanin content in purple (lines 3 and 4) and blue petals (line 5) was significantly higher than in petals of other colors (lines 1 and 2) (Figure 2d, Table 1); flavones and flavonols contents in purple and blue petals were significantly lower than in white color petals (Figure 2d, Table 1, Supplementary Figure S3). The content of anthocyanins accounted for 6.37%, 8.79%, 86.05%, 93.90%, and 93.82% of the total pigment content in lines 1, 2, 3, 4 and 5, respectively. No significant difference was observed in anthocyanin content between lines 1 and 2 (Figure 2d, Table 1). The content of anthocyanins in lines 3, 4, and 5 was 73.53, 122.83, and 117.66 times that of line 1, respectively (Table 1). The content of anthocyanins in lines 4 and 5 was 1.67 and 1.60 times that of line 3, respectively (Table 1). The results indicated that the difference in petal color between white and purple/blue was specifically caused by the difference in anthocyanin content. Thus, anthocyanin biosynthesis is crucial for color heterogeneity in alfalfa.

### 3.3. Delphinidin-3,5-O-Diglucoside Is the Key Pigment Metabolite Affecting the Purple and Blue Color of Alfalfa Flowers

**Figure 3:**
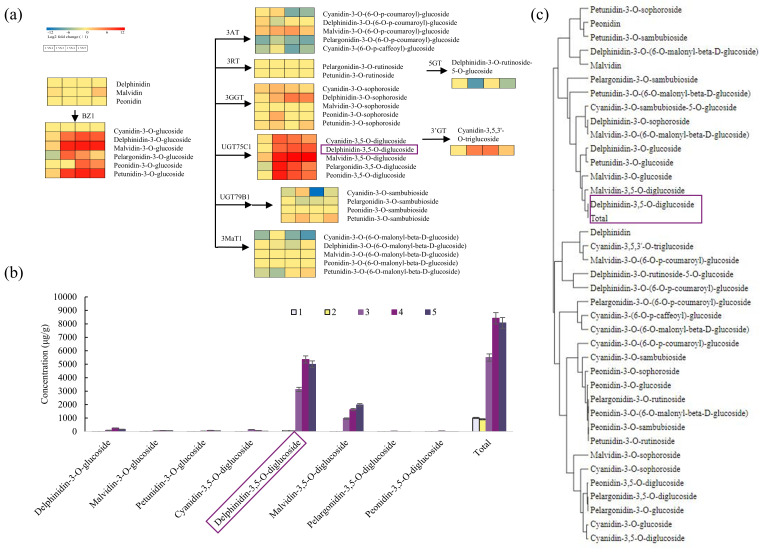
Comparison of metabolites of anthocyanin biosynthesis in alfalfa lines with different petal colors. (**a**) Heatmap of differential content of anthocyanins in alfalfa lines with different petal colors. (**b**) Content of the top eight anthocyanins and the total pigment metabolites in the five alfalfa lines. Error bars represent standard deviations (SDs) of three biological replicates. (**c**) Cluster analysis of anthocyanins and total pigment metabolites. BZ1: anthocyanidin 3-*O*-glucosyltransferase; 3AT: anthocyanidin 3-*O*-glucoside 6″-*O*-acyltransferase; 3RT: anthocyanidin 3-*O*-rutinosyltransferase; 3GGT: anthocyanidin 3-O-glucoside 2″-*O*-glucosyltransferase; UGT75C1: anthocyanidin 3-*O*-glucoside 5-*O*-glucosyltransferase; UGT79B1: anthocyanidin 3-*O*-glucoside 2‴-*O*-xylosyltransferase; 3MaT1: anthocyanin 3-*O*-glucoside-6″-*O*-malonyltransferase; 5GT: cyanidin 3-*O*-rutinoside 5-*O*-glucosyltransferase; 3′GT: anthocyanin 3′-*O*-beta-glucosyltransferase.

According to the basic structure of anthocyanins, a total of 52 anthocyanins were identified in alfalfa petals, including 14 cyanidins, 11 delphinidins, 7 malvidins, 8 pelargonidins, 6 peonidins, and 7 petunidins (Supplementary Table S1). The content of delphinidins accounted for 5.51%, 6.84%, 58.58%, 66.76%, and 64.04% of the total pigment content in lines 1, 2, 3, 4, and 5, respectively (Supplementary Figure S4a). According to the functional groups, 9 types of anthocyanins were detected, including anthocyanidin-3,5-*O*-diglucoside, anthocyanidin-3-*O*-glucoside, anthocyanidin-3-*O*-(6-*O*-p-coumaroyl)-glucoside, anthocyanidin-3-*O*-rutinoside, anthocyanidin-3-*O*-sophoroside, anthocyanidin-3-*O*-sambubioside, anthocyanidin-3-*O*-(6-*O*-malonyl-beta-D-glucoside), delphinidin-3-*O*-rutinoside-5-*O*-glucoside and cyanidin-3,5,3′-*O*-triglucoside. Anthocyanidin-3,5-*O*-diglucoside catalyzed by UGT75C1 had the most significant difference between line 1 and lines 3/4/5. The content of anthocyanidin-3,5-*O*-diglucoside accounted for 5.61%, 7.28%, 78.79%, 84.04%, and 86.74% of the total pigment content in lines 1, 2, 3, 4, and 5, respectively (Figure 3a, Supplementary Figures S4b and S5).

As one of anthocyanidin-3,5-*O*-diglucosides, delphinidin-3,5-*O*-diglucoside content in lines 1 and 2 was only about 5.26% and 6.38% of total pigment, while in lines 3, 4, and 5, the delphinidin-3,5-*O*-diglucoside content was 56.90%, 63.62%, and 61.94% of total pigment, respectively (Figure 3b). The content of delphinidin-3,5-*O*-diglucoside in lines 3, 4, and 5 was 58.88, 100.80, and 94.07 times that of line 1, respectively. Cluster analysis of all anthocyanins and total pigments showed that delphinidin-3,5-*O*-diglucoside was closest to the total pigments (Figure 3c). Therefore, delphinidin-3,5-*O*-diglucoside was the key pigment causing the purple or blue petal color in alfalfa.

### 3.4. Alfalfa Petal Color Is Affected by the Expression of Flavonoid Biosynthesis-Related Pathway Genes

**Figure 4:**
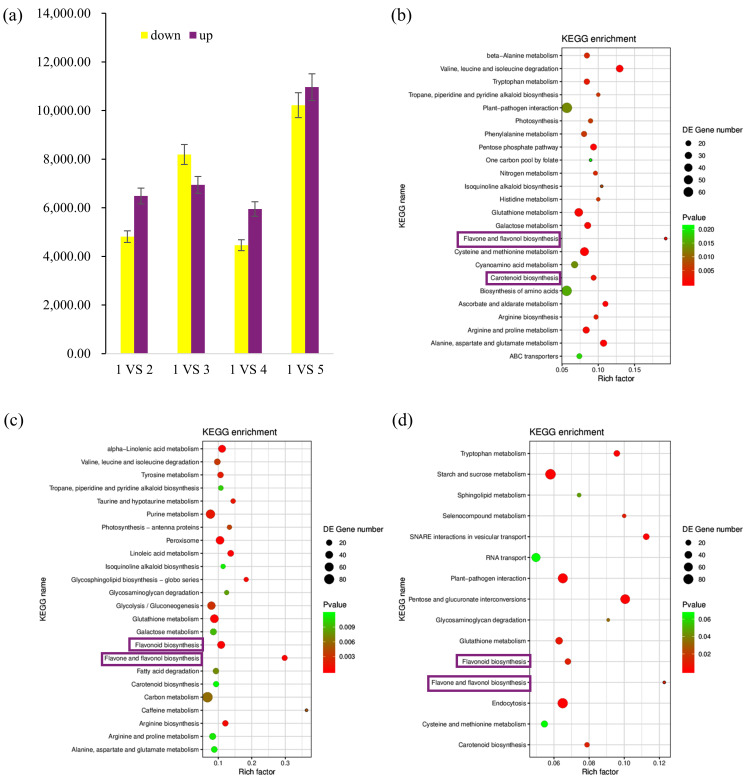
Differentially expressed genes (DEGs) in alfalfa lines with various petal colors. (**a**) DEGs in different comparison groups. Purple indicates upregulated DEGs, and yellow indicates downregulated DEGs. (**b**–**d**) Upregulated DEG enrichment KEGG scatter plot; (**b**) 1 vs. 2 group; (**c**) 1 vs. 3 group; (**d**) 1 vs. 4 group.

RNA-seq analysis was conducted using petals collected from different colored alfalfa lines. More than 40 million raw reads were generated from each library, and 88–93% of the trimmed reads were uniquely mapped to the alfalfa (cultivar “XinJiangDaYe”) reference genome (Supplementary Table S2). The differentially expressed genes (DEGs) were analyzed by DESeq software, and the selected DEGs (|log2FC| ≥ 1 and FDR < 0.05) were then compared and analyzed. Compared to the line 1 control, 11,300, 15,137, 10,414, and 21,186 genes were differentially expressed in lines 2, 3, 4, and 5, respectively, of which 6486, 6942, 5950, and 10,965, respectively, were upregulated and 4814, 8195, 4464, and 10,221, respectively, were downregulated (Figure 4a). In general, there were more upregulated DEGs than downregulated DEGs.

The upregulated DEGs identified in the 1 vs. 2–5 comparison groups were involved in 120–122 KEGG metabolic pathways, and 24, 24, 15, and 23 significantly enriched metabolic pathways were identified in the 1 vs. 2, 1 vs. 3, 1 vs. 4, and 1 vs. 5 groups, respectively. Among them, flavone and flavonol biosynthesis (ko00944) and carotenoid biosynthesis (ko00906) were significantly enriched in 1 vs. 2 (Figure 4b). Flavone and flavonol biosynthesis (ko00944) and flavonoid biosynthesis (ko00941) were all significantly enriched in 1 vs. 3 and 1 vs. 4 (Figure 4c,d). Thus, flavonoid biosynthesis-related pathways may play an important role in petal color.

### 3.5. Analysis of DEGs Related to Flavonoid and Anthocyanin Biosynthesis Pathway

Compared to line 1, 1754 genes were expressed in pathways related to flavonoid biosynthesis. Among them, 212 genes were involved in anthocyanin biosynthesis, 778 genes belonged to the UGT family, and 764 genes related to MBW proteins (439 *MYB* and *MYB*-related genes, 194 *bHLH*, and 131 *WD40*) (Supplementary Figure S6).

**Figure 5:**
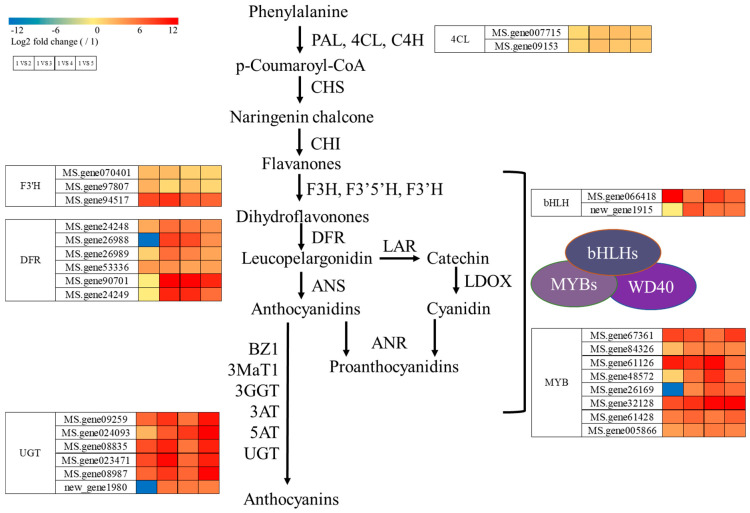
Cluster heatmap of candidate genes. PAL: phenylalanine ammonia-lyase; 4CL: 4-coumarate: CoA ligase; C4H: cinnamate-4-hydroxylase; CHS: chalcone synthase; CHI: chalcone isomerase; F3′H: flavonoid 3′-hydroxylase; F3′H: flavonoid 3′-hydroxylase; F3′5′H: flavonoid 3′,5′-hydroxylase; DFR: dihydroflavonol reductase; LAR: leucoanthocyanidin reductase; ANS: anthocyanidin synthase; LDOX: leucoanthocyanidin dioxygenase; ANR: anthocyanidin reductase; BZ1: anthocyanidin 3-*O*-glucosyltransferase; 3MaT1: anthocyanin 3-*O*-glucoside-6″-*O*-malonyltransferase; 3GGT: anthocyanidin 3-*O*-glucoside 2″-*O*-glucosyltransferase; 3AT: anthocyanidin 3-*O*-glucoside 6″-*O*-acyltransferase; 5AT: anthocyanin 5-aromatic acyltransferase; UGT: UDP-glycosyltransferases.

Based on KEGG enrichment analysis and petal color differences, 27 DEGs (every sample FPKM > 1, |log2FC| ≥ 1 in 1 vs. 3, 1 vs. 4, and 1 vs. 5 groups) were significantly enriched in anthocyanin biosynthesis-related pathways. These 27 DEGs include 2 *4CL*, 3 *F3′H*, 6 *DFR*, 6 *UGT*, 2 *bHLH*, and 8 *MYB* genes, which are considered candidate genes regulating purple petal c−lor in alfalfa (Figure 5, Supplementary Table S3).

### 3.6. Lutein and β-Carotene Are the Main Pigment Metabolites Affecting the Yellow Petal Color in Alfalfa

Finally, we analyzed the cause of the formation of yellow petals in alfalfa. According to RNA-seq analysis, upregulated DGEs were enriched in the carotenoid biosynthesis pathway and flavone and flavonol biosynthesis pathway in the 1 vs. 2 group (Figure 4). Compared to white-colored line 1, the content of flavones and flavonols was significantly lower (16.31% and 16.20%, respectively) in yellow-colored line 2, while the content of total carotenoids was 2.35 times that of line 1 (Table 1). Therefore, carotenoids play a key role in determining the yellow petal color in alfalfa.

**Figure 6:**
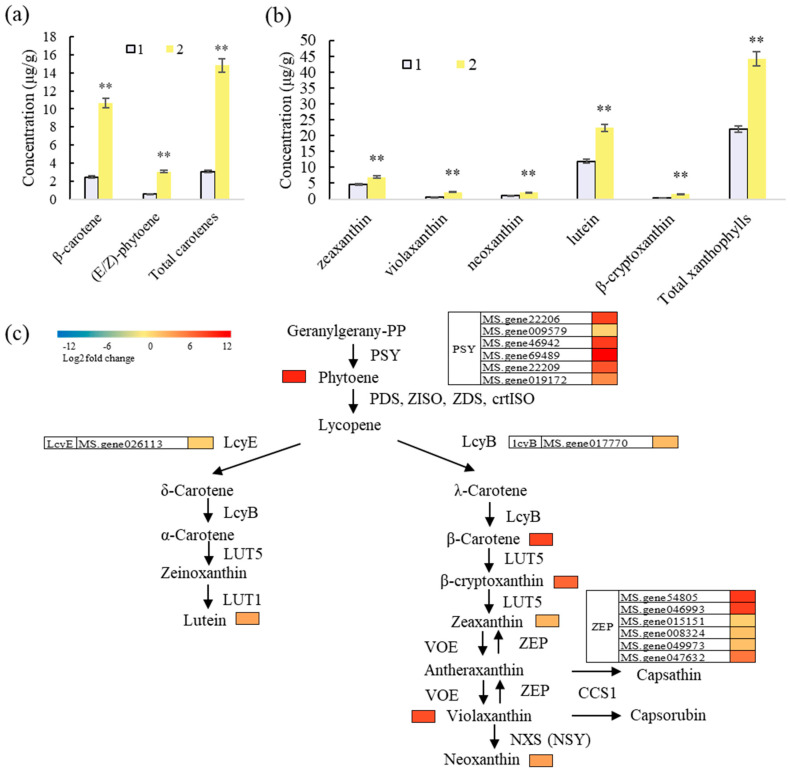
Yellow petal formation is associated with carotenoids. (**a**) Main carotene content. (**b**) Main xanthophyll content. Error bars represent standard deviations (SDs) of three biological replicates. ** stand for *p* < 0.01. (**c**) Heatmap of differential content of carotenoids in alfalfa lines with different petal colors. PSY: phytoene synthase; PDS: phytoene desaturase; ZISO: zeta-carotene isomerase; ZDS: zeta-carotene desaturase; crtISO: prolycopene isomerase; LcyE: lycopene epsilon cyclase; LcyB: lycopene beta cyclase; LUT5: beta-ring hydroxylase; LUT1: carotenoid epsilon hydroxylase; ZEP: zeaxanthin epoxidase; VDE: violaxanthin de-epoxidase; NXS (NSY): neoxanthin synthase; CCS1: capsanthin/capsorubin synthase.

Compared to line 1, the top seven carotenoids (lutein, β-carotene, zeaxanthin, (E/Z)-phytoene, violaxanthin, neoxanthin, and β-cryptoxanthin) were significantly higher in line 2 (Figure 6a,b). In the carotene category, the content of β-carotene was the highest, accounting for 18.05% of total carotenoids (total carotene + total xanthophylls) in line 2, and it was 4.29 times that of line 1 (Figure 6a). Among the xanthophylls, lutein content was the highest, accounting for 38.04% of total carotenoids in line 2, and it was 1.89 times that of line 1 (Figure 6b). Therefore, lutein and β-carotene were the main metabolites leading to the yellow petal color in alfalfa. Compared to line 1, 6 *PSY*, 1 *LcyE*, 1 *LcyB*, and 6 *ZEP* genes were significantly upregulated in line 2 and likely to be involved in regulating the formation of yellow petals in alfalfa (Figure 6c).

## 4. Discussion

Variation in flower color plays an important ecological role by attracting pollinators and influencing reproductive success in flowering plants, particularly for plants with a high degree of self-incompatibility, such as alfalfa. Alfalfa is a widely cultivated forage crop around the world due to its remarkable adaptability, high biomass yield, exceptional nutritive value, and notable capacity for nitrogen fixation [23,26,27]. Although various flower colors have been observed and described in alfalfa a long time ago [28], the biochemical and molecular mechanism of its color formation is still unclear. By analyzing alfalfa lines with five contrasting flower colors, various kinds and levels of anthocyanins, carotenoids and other flavonoids were detected in different colored petals, and their roles in color formation were revealed. Genes responsible for specific biochemical pathways and compounds associated with color formation were also revealed. This study provides an in-depth interpretation of the flower coloration mechanism in alfalfa.

### 4.1. Key Metabolic Compounds Affecting Petal Color Formation in Alfalfa

The presence of specific types of anthocyanins affects flower colors. For example, cyanidin 3-glucoside and cyanidin 3-(6-malonyglucoside) are dominant pigments in the black-colored flowers in the Alpine orchid *Gymnadenia rhellicani* [29]; maintaining both high relative and absolute content of cyanidin 3,5-*O*-diglucoside may be a prerequisite for the formation of red petals in *Rosa rugosa* [30].

Various types of anthocyanins have been reported in the formation of purple and blue flowers. Cyanidin 3,5-*O*-diglucoside, malvidin 3,5-diglucoside, and cyanidin 3-*O*-galactoside were found to be responsible for the purple flower color in *Salvia miltiorrhiza* [31]. High levels of malvidin-3,5-di-*O*-glucoside were detected in purple flowers in *Lagerstroemia indica* [32]. Significantly higher content of delphinidin-3,5-*O*-diglucoside was detected in 8–16 deep purple–red-flowered germplasm resources with somewhat unique and visible blue hue in *R. rugosa* [30].

In the current study, we found that delphinidin-3,5-*O*-diglucoside is the primary anthocyanin responsible for the formation of both purple and blue colored petals in alfalfa. This conclusion is consistent with the work of Wang et al. in *R. rugosa* [30], but different from that of Duan et al. in alfalfa [33], which used only two different colored lines.

The formation of yellow flowers is considered to come from flavonoids or carotenoids. The yellow-colored tree peony flower displayed relatively higher contents of tetrahydroxychalcone (THC), flavones, and flavonols compared to purple–red flowers, but no anthocyanin production was detected [34]. More flavonoid metabolic compounds were detected in yellow and white flowers of safflower compared to red flowers [35]. All-*trans*-violaxanthin and total carotenoid were the main factors affecting yellow flower color in Narcissus [36]. In the current study, we found that lutein and β-carotene are the main factors contributing to the formation of yellow-colored petals in alfalfa.

### 4.2. Key Candidate Genes Responsible for Petal Color Formation in Alfalfa

The flavonoid synthesis pathway is relatively conserved, and it has been well studied in *Arabidopsis* and other model plants [16,37]. However, the transcription levels of structural genes and transcription factors involved in the anthocyanin biosynthesis pathway differ among plants or in the same plant at different developmental stages or in different tissues, thus affecting the formation of flower colors. In *Cymbidium lowianum*, the red coloration flower is correlated with high levels of *F3′H* expression compared to the albino flower [38]. In the current study, genes involved in the anthocyanin biosynthesis pathway were identified in alfalfa, including enzymatic genes *4CL* (2), *F3′H* (3), *DFR* (6), and *UGT* (6), and transcription factor genes *bHLH* (2) and *MYB* (8).

Differential expression of an *ANS* gene regulated by *MYB1* affects the morphs that differ solely in cyanidin pigments, making black, red, and white floral morphs in the Alpine orchid [29]. *CgsMYB12* is an R2R3-MYB transcription factor responsible for anthocyanin pigmentation of the basal region (‘cup’) in the petal of *C. gracilis ssp. Sonomensis* [39]. In *Chrysanthemum morifolium*, the difference in petal color is caused by the expression variation in the anthocyanin biosynthesis gene *CmMYB6*; after demethylation of the *CmMYB6* promoter using the dCas9-TET1cd system, the flower color returns from yellow to pink [40]. Overexpression of *CsMYB5-1* and *CsMYB5-2* slightly lightens the color of the flowers in alfalfa [41]. MYBs are also involved in carotenoid accumulation. WHITE PETAL1 (WP1) is an anthocyanin-related R2R3-MYB protein that plays a critical role in regulating floral carotenoid pigmentation in *Medicago truncatula* [42]. In the current study, eight *MYBs* (*MS.gene67361*, *MS.gene84326*, *MS.gene61126*, *MS.gene48572*, *MS.gene26169*, *MS.gene32128*, *MS.gene61428*, and *MS.gene005866*) were identified to be involved in the formation of purple petals. These *MYB* genes, together with the enzymatic genes and the *bHLH* genes, play important roles in color formation in alfalfa flowers based on RNA-seq, but their specific functions, especially in relation to colors, need to be studied further.

## 5. Conclusions

**Figure 7:**
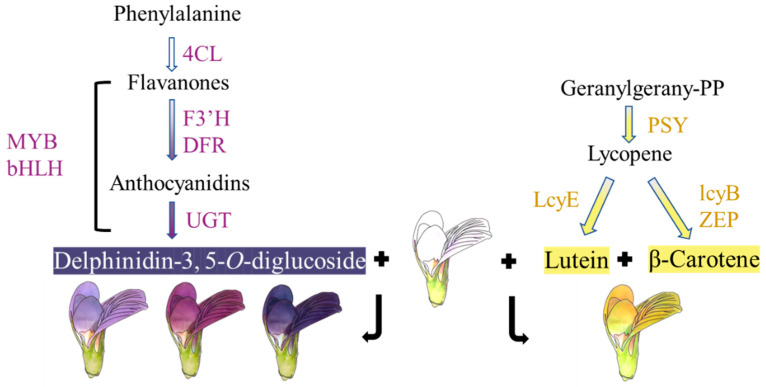
Schematic mechanism of flower color formation in alfalfa. 4CL: 4-coumarate: CoA ligase; F3′H: flavonoid 3′-hydroxylase; DFR: dihydroflavonol reductase; UGT: UDP-glycosyltransferases; PSY: phytoene synthase; LcyE: lycopene epsilon cyclase; LcyB: lycopene beta cyclase; ZEP: zeaxanthin epoxidase.

In this article, we used metabolic profiling and RNA-seq to explain the formation of alfalfa heterochromatic flowers. The difference in petal color between white (line 1), purple (lines 3 and 4), and blue (line 5) petals was mainly caused by the difference in *4CL*, *F3′H*, *DFR*, *UGT*, *bHLH*, and *MYB* expression levels and consequently the accumulation of delphinidin-3,5-O-diglucoside (Figure 7). The difference in petal color between white (line 1) and yellow (line 2) petals was mainly affected by the genes *PSY*, *LcyE*, *lcyB*, and *ZEP*, and consequently the production of lutein and β-carotene, resulting in the change in flower color from white to yellow (Figure 7).

## Figures and Tables

**Table 1:** Pigment content in alfalfa lines (1–5) with different petal colors. Groups with significant differences are labeled with different letters, while groups without significant differences are labeled with the same letter. Lowercase letters represent *p* < 0.05, while uppercase letters represent *p* < 0.01.differences. Capital letters represent significant differences, while lowercase letters represent significant differences.

	Anthocyanidins	Flavones	Flavonols	Carotenoids	Flavanols	Procyanidins	Xanthophylls	Phenonic Acids	Isoflavanones	Chalcones	Total Carotenoids
**1**	64.33 C d	482.82 A a	433.98 A a	3.09 E e	0.38 C c	1.6 × 10^2^ A a	22.02 C c	0.17 C d	1.02 AB a	2.54 C c	25.11 C e
**2**	79.99 C d	404.08 B b	363.68 B b	14.81 A a	0.17 D d	9.56 × 10^3^ C cd	44.13 A a	0.30 A a	0.54 C d	2.17 D d	58.94 A a
**3**	4730.31 B c	394.62 B b	339.02 B b	3.52 D d	1.02 B b	1.26 × 10^2^ AB b	24.04 C c	0.18 C d	0.91 B b	3.42 B b	27.55 C d
**4**	7902.30 A a	206.55 C c	252.62 C c	5.72 C c	1.95 A a	7.44 × 10^3^ C d	41.27 AB b	0.25 B b	1.07A a	4.28 A a	46.99 B c
**5**	7569.34 A b	167.05 D d	275.39 C c	13.41 B b	0.99 B b	1.12 × 10^2^ BC bc	39.26 B b	0.22 B c	0.65 C c	1.46 E e	52.68 AB b

## Data Availability

The datasets used and/or analyzed during the current study are included in this article and available from the corresponding author upon reasonable request. The sequenced raw reads generated in this study have been submitted to the National Center for Biotechnology Information (NCBI) with BioProject ID: PRJNA1108429.

## Supplementary Materials

The following supporting information can be downloaded at: https://www.mdpi.com/article/10.3390/metabo15020135/s1. Supplementary (pptx): Table S1, Anthocyanidin content in alfalfa lines with different petal colors; Table S2, Comparison statistics of the filtered RNA and the reference genome; Table S3, Details of candidate genes; Figure S1: Style, stigma, ovary, filament, and anther color of different alfalfa lines; Figure S2, Scanning electron microscope (SEM) of alfalfa lines with different flower colors; Figure S3, Content of different pigment; Figure S4, Content of different anthocyanin types; Figure S5, Content of different anthocyanin types; Figure S6, Cluster heatmap of all expressed genes inanthocyanin biosynthetic pathway in alfalfa.

## References

[1] Gigord L.D., Macnair M.R., Smithson A. (2001). Negative frequency-dependent selection maintains a dramatic flower color polymorphism in the rewardless orchid *Dactylorhiza sambucina* (L.) Soo. Proc. Natl. Acad. Sci. USA.

[2] Guitián J.A., Sobral M., Veiga T., Losada M., Guitián P., Guitián J.M. (2017). Differences in pollination success between local and foreign flower color phenotypes: A translocation experiment with *Gentiana lutea* (Gentianaceae). PeerJ.

[3] Zhou Z., Ying Z., Wu Z., Yang Y., Fu S., Xu W., Yao L., Zeng A., Huang J., Lan S. (2021). Anthocyanin genes involved in the glower coloration mechanisms of *Cymbidium kanran*. Front. Plant Sci..

[4] Chen H., Zeng Y., Yang Y., Huang L., Tang B., Zhang H., Hao F., Liu W., Li Y., Liu Y. (2020). Allele-aware chromosome-level genome assembly and efficient transgene-free genome editing for the autotetraploid cultivated alfalfa. Nat. Commun..

[5] Shen C., Du H., Chen Z., Lu H., Zhu F., Chen H., Meng X., Liu Q., Liu P., Zheng L. (2020). The chromosome-level genome sequence of the autotetraploid alfalfa and resequencing of core germplasms provide genomic resources for alfalfa research. Mol. Plant.

[6] Brunet J., Flick A.J., Bauer A.A. (2020). Phenotypic selection on flower color and floral display size by three bee species. Front. Plant Sci..

[7] Ehlting J., Büttner D., Wang Q., Douglas C.J., Somssich I.E., Kombrink E. (1999). Three 4-coumarate:coenzyme A ligases in *Arabidopsis thaliana* represent two evolutionarily divergent classes in angiosperms. Plant J..

[8] Saslowsky D., Winkel-Shirley B. (2001). Localization of flavonoid enzymes in *Arabidopsis* roots. Plant J..

[9] Moreau C., Ambrose M.J., Turner L., Hill L., Ellis T.H.N., Hofer J.M.I. (2012). The *b* gene of pea encodes a defective flavonoid 3′,5′-hydroxylase, and confers pink flower color. Plant Physiol..

[10] Shirley B.W., Kubasek W.L., Storz G., Bruggemann E., Koornneef M., Ausubel F.M., Goodman H.M. (1995). Analysis of *Arabidopsis* mutants deficient in flavonoid biosynthesis. Plant J..

[11] Debeaujon I., Léon-Kloosterziel K.M., Koornneef M. (2000). Influence of the testa on seed dormancy, germination, and longevity in *Arabidopsis*. Plant Physiol..

[12] Baudry A., Heim M.A., Dubreucq B., Caboche M., Weisshaar B., Lepiniec L. (2004). TT2, TT8, and TTG1 synergistically specify the expression of *BANYULS* and proanthocyanidin biosynthesis in *Arabidopsis thaliana*. Plant J..

[13] Nesi N., Debeaujon I., Jond C., Pelletier G., Caboche M., Lepiniec L. (2000). The *TT8* gene encodes a basic helix-loop-helix domain protein required for expression of *DFR* and *BAN* genes in *Arabidopsis* siliques. Plant Cell.

[14] Zhang F., Gonzalez A., Zhao M., Payne C.T., Lloyd A. (2003). A network of redundant bHLH proteins functions in all TTG1-dependent pathways of *Arabidopsis*. Development.

[15] Albert N.W., Davies K.M., Lewis D.H., Zhang H., Montefiori M., Brendolise C., Boase M.R., Ngo H., Jameson P.E., Schwinn K.E. (2014). A conserved network of transcriptional activators and repressors regulates anthocyanin pigmentation in eudicots. Plant Cell.

[16] Lepiniec L., Debeaujon I., Routaboul J.M., Baudry A., Pourcel L., Nesi N., Caboche M. (2006). Genetics and biochemistry of seed flavonoids. Annu. Rev. Plant Biol..

[17] Li P., Chen B., Zhang G., Chen L., Dong Q., Wen J., Mysore K.S., Zhao J. (2016). Regulation of anthocyanin and proanthocyanidin biosynthesis by *Medicago truncatula* bHLH transcription factor MtTT8. New Phytol..

[18] Chai Q., Wang X., Gao M., Zhao X., Chen Y., Zhang C., Jiang H., Wang J., Wang Y., Zheng M. (2023). A glutathione S-transferase GhTT19 determines flower petal pigmentation via regulating anthocyanin accumulation in cotton. Plant Biotechnol. J..

[19] Kong J.M., Chia L.S., Goh N.K., Chia T.F., Brouillard R. (2003). Analysis and biological activities of anthocyanins. Phytochemistry.

[20] Jaakola L. (2013). New insights into the regulation of anthocyanin biosynthesis in fruits. Trends Plant Sci..

[21] Wang Y., Li X., Qiu H., Chen R., Xiong A., Xu Z., Miao W., Chen R., Wang P., Hou X. (2025). The MADS-RIPENING INHIBITOR–DIVARICATA1 module regulates carotenoid biosynthesis in nonclimacteric Capsicum fruits. Plant Physiol..

[22] Peng A., Tang X., Feng Y., Huang Y., Cui J., Tian K., Lu M., Zhao Y., Pan Y., Wang S. (2023). Molecular mechanism of lycopene cyclases regulating carotenoids ratio in different branches during tea leaf and flower development. Hortic. Plant J..

[23] Gou J., Debnath S., Sun L., Flanagan A., Tang Y., Jiang Q., Wen J., Wang Z.Y. (2017). From model to crop: Functional characterization of SPL8 in *M. truncatula* led to genetic improvement of biomass yield and abiotic stress tolerance in alfalfa. Plant Biotechnol. J..

[24] Kim D., Langmead B., Salzberg S.L. (2015). HISAT: A fast spliced aligner with low memory requirements. Nat. Methods.

[25] Xie C., Mao X., Huang J., Ding Y., Wu J., Dong S., Kong L., Gao G., Li C.Y., Wei L. (2011). KOBAS 2.0: A web server for annotation and identification of enriched pathways and diseases. Nucleic Acids Res..

[26] Annicchiarico P., Nazzicari N., Li X., Wei Y., Pecetti L., Brummer E.C. (2015). Accuracy of genomic selection for alfalfa biomass yield in different reference populations. BMC Genom..

[27] Aung B., Gruber M.Y., Amyot L., Omari K., Bertrand A., Hannoufa A. (2015). MicroRNA156 as a promising tool for alfalfa improvement. Plant Biotechnol. J..

[28] Barnes D.K. (1972). A System for Visually Classifying Alfalfa Flower Color.

[29] Kellenberger R.T., Byers K.J.R.P., De Brito Francisco R.M., Staedler Y.M., LaFountain A.M., Schönenberger J., Schiestl F.P., Schlüter P.M. (2019). Emergence of a floral colour polymorphism by pollinator-mediated overdominance. Nat. Commun..

[30] Wang Y., Li S., Zhu Z., Xu Z., Qi S., Xing S., Yu Y., Wu Q. (2022). Transcriptome and chemical analyses revealed the mechanism of flower color formation in *Rosa rugosa*. Front. Plant Sci..

[31] Jiang T., Zhang M., Wen C., Xie X., Tian W., Wen S., Lu R., Liu L. (2020). Integrated metabolomic and transcriptomic analysis of the anthocyanin regulatory networks in *Salvia miltiorrhiza* Bge. flowers. BMC Plant Biol..

[32] Hong S., Wang J., Wang Q., Zhang G., Zhao Y., Ma Q., Wu Z., Ma J., Gu C. (2022). Decoding the formation of diverse petal colors of *Lagerstroemia indica* by integrating the data from transcriptome and metabolome. Front. Plant Sci..

[33] Duan H.-R., Wang L.-R., Cui G.-X., Zhou X.-H., Duan X.-R., Yang H.-S. (2020). Identification of the regulatory networks and hub genes controlling alfalfa floral pigmentation variation using RNA-sequencing analysis. BMC Plant Biol..

[34] Luo X., Sun D., Wang S., Luo S., Fu Y., Niu L., Shi Q., Zhang Y. (2021). Integrating full-length transcriptomics and metabolomics reveals the regulatory mechanisms underlying yellow pigmentation in tree peony (*Paeonia suffruticosa* Andr.) flowers. Hortic. Res..

[35] Wang R., Ren C., Dong S., Chen C., Xian B., Wu Q., Wang J., Pei J., Chen J. (2021). Integrated metabolomics and transcriptome analysis of flavonoid biosynthesis in safflower (*Carthamus tinctorius* L.) with different colors. Front. Plant Sci..

[36] Gómez-Gómez L., Li X., Lu M., Tang D., Shi Y. (2015). Composition of carotenoids and flavonoids in Narcissus cultivars and their relationship with flower color. PLoS ONE.

[37] Jun J.H., Xiao X., Rao X., Dixon R.A. (2018). Proanthocyanidin subunit composition determined by functionally diverged dioxygenases. Nat. Plants.

[38] Dong X.M., Zhang W., Tu M., Zhang S.B. (2025). Spatial and temporal regulation of flower coloration in *Cymbidium lowianum*. Plant Cell Environ..

[39] Lin R.C., Rausher M.D. (2021). *R2R3-MYB* genes control petal pigmentation patterning in *Clarkia gracilis* ssp. sonomensis (Onagraceae). New Phytol..

[40] Tang M., Xue W., Li X., Wang L., Wang M., Wang W., Yin X., Chen B., Qu X., Li J. (2022). Mitotically heritable epigenetic modifications of *CmMYB6* control anthocyanin biosynthesis in chrysanthemum. New Phytol..

[41] Zhang H., Zheng G., Fan C., Di S., Wang X., Gao L., Dzyubenko N., Chapurin V., Pang Y. (2019). Ectopic expression of tea *MYB* genes alter spatial flavonoid accumulation in alfalfa (*Medicago sativa*). PLoS ONE.

[42] Meng Y., Wang Z., Wang Y., Wang C., Zhu B., Liu H., Ji W., Wen J., Chu C., Tadege M. (2019). The MYB activator WHITE PETAL1 associates with MtTT8 and MtWD40-1 to regulate carotenoid-derived flower pigmentation in *Medicago truncatula*. Plant Cell.

