# Supplementary material for: Integrated Analyses of the Mechanism of Flower Color Formation in Alfalfa (Medicago sativa)
Source: Metabolites. 2025 Feb 17;15(2):135. doi: 10.3390/metabo15020135 (PMC11857827; doi:10.3390/metabo15020135)
Supplement: Supplementary file 1 [file metabolites-15-00135-s001.zip › metabolites-3437683-supplementary.pptx]

## Slide 1
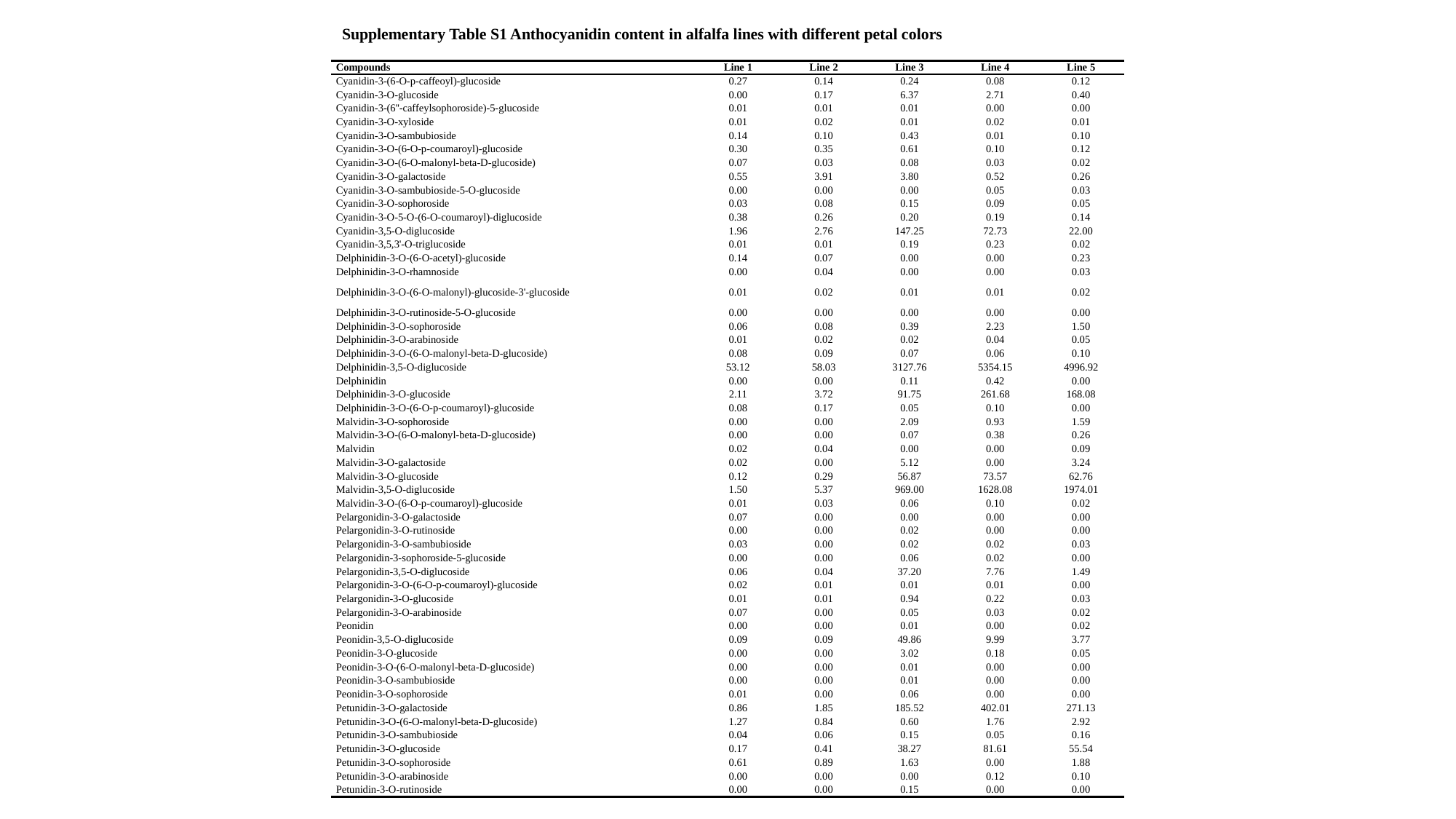

Supplementary Table S1 Anthocyanidin content in alfalfa lines with different petal colors
| Compounds | Line 1 | Line 2 | Line 3 | Line 4 | Line 5 |
| --- | --- | --- | --- | --- | --- |
| Cyanidin-3-(6-O-p-caffeoyl)-glucoside | 0.27 | 0.14 | 0.24 | 0.08 | 0.12 |
| Cyanidin-3-O-glucoside | 0.00 | 0.17 | 6.37 | 2.71 | 0.40 |
| Cyanidin-3-(6''-caffeylsophoroside)-5-glucoside | 0.01 | 0.01 | 0.01 | 0.00 | 0.00 |
| Cyanidin-3-O-xyloside | 0.01 | 0.02 | 0.01 | 0.02 | 0.01 |
| Cyanidin-3-O-sambubioside | 0.14 | 0.10 | 0.43 | 0.01 | 0.10 |
| Cyanidin-3-O-(6-O-p-coumaroyl)-glucoside | 0.30 | 0.35 | 0.61 | 0.10 | 0.12 |
| Cyanidin-3-O-(6-O-malonyl-beta-D-glucoside) | 0.07 | 0.03 | 0.08 | 0.03 | 0.02 |
| Cyanidin-3-O-galactoside | 0.55 | 3.91 | 3.80 | 0.52 | 0.26 |
| Cyanidin-3-O-sambubioside-5-O-glucoside | 0.00 | 0.00 | 0.00 | 0.05 | 0.03 |
| Cyanidin-3-O-sophoroside | 0.03 | 0.08 | 0.15 | 0.09 | 0.05 |
| Cyanidin-3-O-5-O-(6-O-coumaroyl)-diglucoside | 0.38 | 0.26 | 0.20 | 0.19 | 0.14 |
| Cyanidin-3,5-O-diglucoside | 1.96 | 2.76 | 147.25 | 72.73 | 22.00 |
| Cyanidin-3,5,3'-O-triglucoside | 0.01 | 0.01 | 0.19 | 0.23 | 0.02 |
| Delphinidin-3-O-(6-O-acetyl)-glucoside | 0.14 | 0.07 | 0.00 | 0.00 | 0.23 |
| Delphinidin-3-O-rhamnoside | 0.00 | 0.04 | 0.00 | 0.00 | 0.03 |
| Delphinidin-3-O-(6-O-malonyl)-glucoside-3'-glucoside | 0.01 | 0.02 | 0.01 | 0.01 | 0.02 |
| Delphinidin-3-O-rutinoside-5-O-glucoside | 0.00 | 0.00 | 0.00 | 0.00 | 0.00 |
| Delphinidin-3-O-sophoroside | 0.06 | 0.08 | 0.39 | 2.23 | 1.50 |
| Delphinidin-3-O-arabinoside | 0.01 | 0.02 | 0.02 | 0.04 | 0.05 |
| Delphinidin-3-O-(6-O-malonyl-beta-D-glucoside) | 0.08 | 0.09 | 0.07 | 0.06 | 0.10 |
| Delphinidin-3,5-O-diglucoside | 53.12 | 58.03 | 3127.76 | 5354.15 | 4996.92 |
| Delphinidin | 0.00 | 0.00 | 0.11 | 0.42 | 0.00 |
| Delphinidin-3-O-glucoside | 2.11 | 3.72 | 91.75 | 261.68 | 168.08 |
| Delphinidin-3-O-(6-O-p-coumaroyl)-glucoside | 0.08 | 0.17 | 0.05 | 0.10 | 0.00 |
| Malvidin-3-O-sophoroside | 0.00 | 0.00 | 2.09 | 0.93 | 1.59 |
| Malvidin-3-O-(6-O-malonyl-beta-D-glucoside) | 0.00 | 0.00 | 0.07 | 0.38 | 0.26 |
| Malvidin | 0.02 | 0.04 | 0.00 | 0.00 | 0.09 |
| Malvidin-3-O-galactoside | 0.02 | 0.00 | 5.12 | 0.00 | 3.24 |
| Malvidin-3-O-glucoside | 0.12 | 0.29 | 56.87 | 73.57 | 62.76 |
| Malvidin-3,5-O-diglucoside | 1.50 | 5.37 | 969.00 | 1628.08 | 1974.01 |
| Malvidin-3-O-(6-O-p-coumaroyl)-glucoside | 0.01 | 0.03 | 0.06 | 0.10 | 0.02 |
| Pelargonidin-3-O-galactoside | 0.07 | 0.00 | 0.00 | 0.00 | 0.00 |
| Pelargonidin-3-O-rutinoside | 0.00 | 0.00 | 0.02 | 0.00 | 0.00 |
| Pelargonidin-3-O-sambubioside | 0.03 | 0.00 | 0.02 | 0.02 | 0.03 |
| Pelargonidin-3-sophoroside-5-glucoside | 0.00 | 0.00 | 0.06 | 0.02 | 0.00 |
| Pelargonidin-3,5-O-diglucoside | 0.06 | 0.04 | 37.20 | 7.76 | 1.49 |
| Pelargonidin-3-O-(6-O-p-coumaroyl)-glucoside | 0.02 | 0.01 | 0.01 | 0.01 | 0.00 |
| Pelargonidin-3-O-glucoside | 0.01 | 0.01 | 0.94 | 0.22 | 0.03 |
| Pelargonidin-3-O-arabinoside | 0.07 | 0.00 | 0.05 | 0.03 | 0.02 |
| Peonidin | 0.00 | 0.00 | 0.01 | 0.00 | 0.02 |
| Peonidin-3,5-O-diglucoside | 0.09 | 0.09 | 49.86 | 9.99 | 3.77 |
| Peonidin-3-O-glucoside | 0.00 | 0.00 | 3.02 | 0.18 | 0.05 |
| Peonidin-3-O-(6-O-malonyl-beta-D-glucoside) | 0.00 | 0.00 | 0.01 | 0.00 | 0.00 |
| Peonidin-3-O-sambubioside | 0.00 | 0.00 | 0.01 | 0.00 | 0.00 |
| Peonidin-3-O-sophoroside | 0.01 | 0.00 | 0.06 | 0.00 | 0.00 |
| Petunidin-3-O-galactoside | 0.86 | 1.85 | 185.52 | 402.01 | 271.13 |
| Petunidin-3-O-(6-O-malonyl-beta-D-glucoside) | 1.27 | 0.84 | 0.60 | 1.76 | 2.92 |
| Petunidin-3-O-sambubioside | 0.04 | 0.06 | 0.15 | 0.05 | 0.16 |
| Petunidin-3-O-glucoside | 0.17 | 0.41 | 38.27 | 81.61 | 55.54 |
| Petunidin-3-O-sophoroside | 0.61 | 0.89 | 1.63 | 0.00 | 1.88 |
| Petunidin-3-O-arabinoside | 0.00 | 0.00 | 0.00 | 0.12 | 0.10 |
| Petunidin-3-O-rutinoside | 0.00 | 0.00 | 0.15 | 0.00 | 0.00 |

## Slide 2
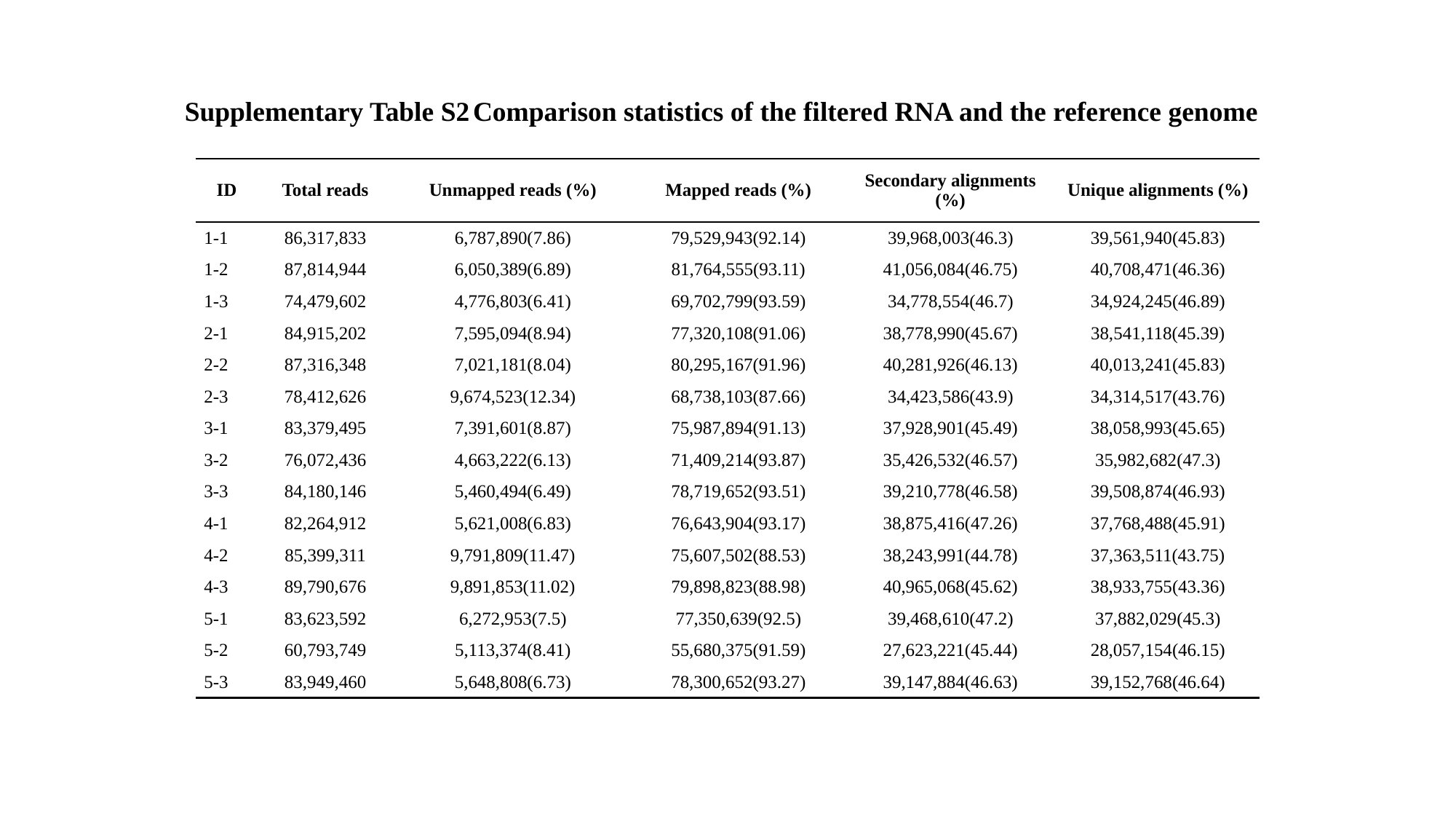

Supplementary Table S2 Comparison statistics of the filtered RNA and the reference genome
| ID | Total reads | Unmapped reads (%) | Mapped reads (%) | Secondary alignments (%) | Unique alignments (%) |
| --- | --- | --- | --- | --- | --- |
| 1-1 | 86,317,833 | 6,787,890(7.86) | 79,529,943(92.14) | 39,968,003(46.3) | 39,561,940(45.83) |
| 1-2 | 87,814,944 | 6,050,389(6.89) | 81,764,555(93.11) | 41,056,084(46.75) | 40,708,471(46.36) |
| 1-3 | 74,479,602 | 4,776,803(6.41) | 69,702,799(93.59) | 34,778,554(46.7) | 34,924,245(46.89) |
| 2-1 | 84,915,202 | 7,595,094(8.94) | 77,320,108(91.06) | 38,778,990(45.67) | 38,541,118(45.39) |
| 2-2 | 87,316,348 | 7,021,181(8.04) | 80,295,167(91.96) | 40,281,926(46.13) | 40,013,241(45.83) |
| 2-3 | 78,412,626 | 9,674,523(12.34) | 68,738,103(87.66) | 34,423,586(43.9) | 34,314,517(43.76) |
| 3-1 | 83,379,495 | 7,391,601(8.87) | 75,987,894(91.13) | 37,928,901(45.49) | 38,058,993(45.65) |
| 3-2 | 76,072,436 | 4,663,222(6.13) | 71,409,214(93.87) | 35,426,532(46.57) | 35,982,682(47.3) |
| 3-3 | 84,180,146 | 5,460,494(6.49) | 78,719,652(93.51) | 39,210,778(46.58) | 39,508,874(46.93) |
| 4-1 | 82,264,912 | 5,621,008(6.83) | 76,643,904(93.17) | 38,875,416(47.26) | 37,768,488(45.91) |
| 4-2 | 85,399,311 | 9,791,809(11.47) | 75,607,502(88.53) | 38,243,991(44.78) | 37,363,511(43.75) |
| 4-3 | 89,790,676 | 9,891,853(11.02) | 79,898,823(88.98) | 40,965,068(45.62) | 38,933,755(43.36) |
| 5-1 | 83,623,592 | 6,272,953(7.5) | 77,350,639(92.5) | 39,468,610(47.2) | 37,882,029(45.3) |
| 5-2 | 60,793,749 | 5,113,374(8.41) | 55,680,375(91.59) | 27,623,221(45.44) | 28,057,154(46.15) |
| 5-3 | 83,949,460 | 5,648,808(6.73) | 78,300,652(93.27) | 39,147,884(46.63) | 39,152,768(46.64) |

## Slide 3
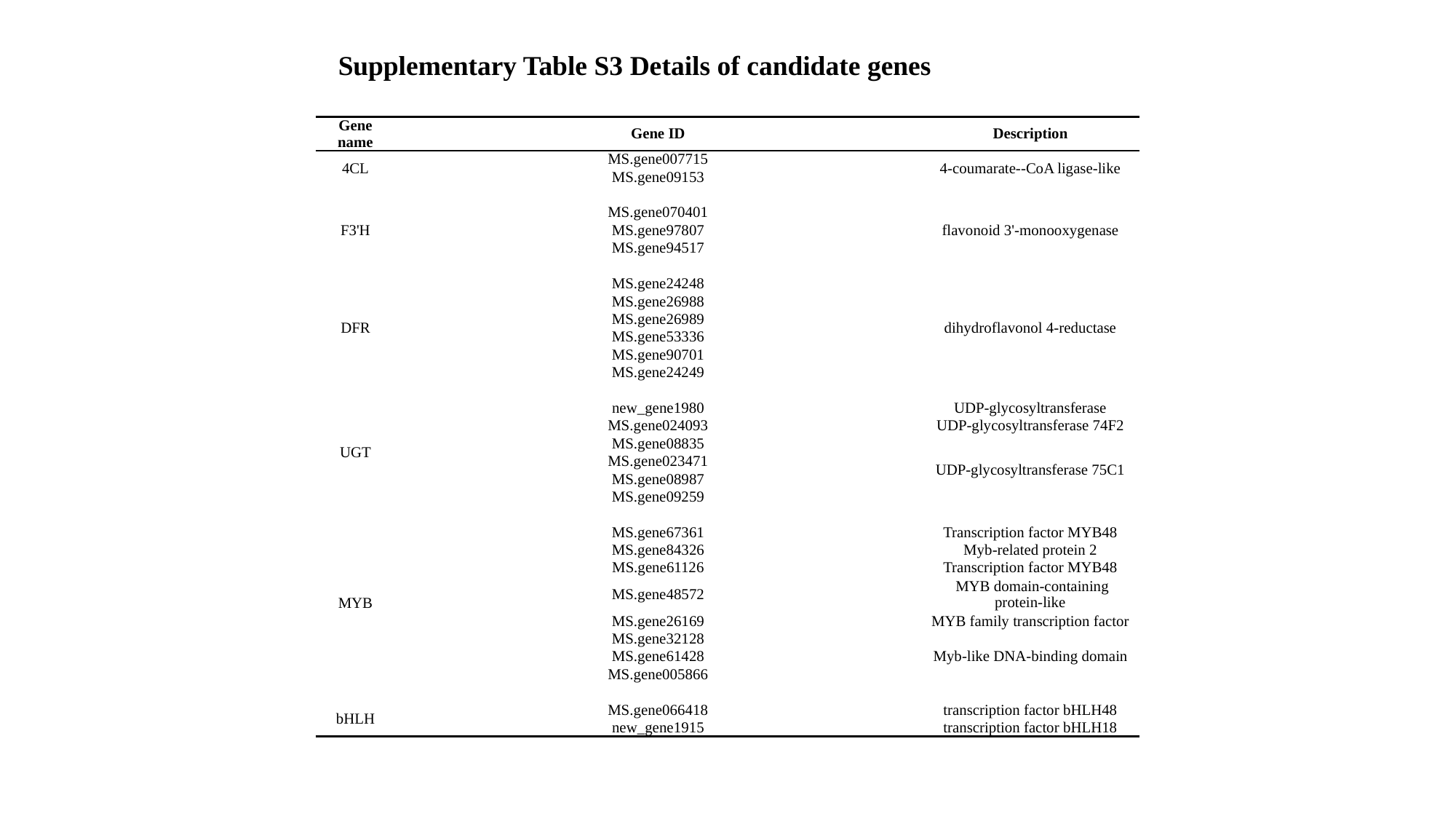

Supplementary Table S3 Details of candidate genes
| Gene name | Gene ID | Description |
| --- | --- | --- |
| 4CL | MS.gene007715 | 4-coumarate--CoA ligase-like |
| | MS.gene09153 | |
| | | |
| F3'H | MS.gene070401 | flavonoid 3'-monooxygenase |
| | MS.gene97807 | |
| | MS.gene94517 | |
| | | |
| DFR | MS.gene24248 | dihydroflavonol 4-reductase |
| | MS.gene26988 | |
| | MS.gene26989 | |
| | MS.gene53336 | |
| | MS.gene90701 | |
| | MS.gene24249 | |
| | | |
| UGT | new\_gene1980 | UDP-glycosyltransferase |
| | MS.gene024093 | UDP-glycosyltransferase 74F2 |
| | MS.gene08835 | UDP-glycosyltransferase 75C1 |
| | MS.gene023471 | |
| | MS.gene08987 | |
| | MS.gene09259 | |
| | | |
| MYB | MS.gene67361 | Transcription factor MYB48 |
| | MS.gene84326 | Myb-related protein 2 |
| | MS.gene61126 | Transcription factor MYB48 |
| | MS.gene48572 | MYB domain-containing protein-like |
| | MS.gene26169 | MYB family transcription factor |
| | MS.gene32128 | Myb-like DNA-binding domain |
| | MS.gene61428 | |
| | MS.gene005866 | |
| | | |
| bHLH | MS.gene066418 | transcription factor bHLH48 |
| | new\_gene1915 | transcription factor bHLH18 |

## Slide 4
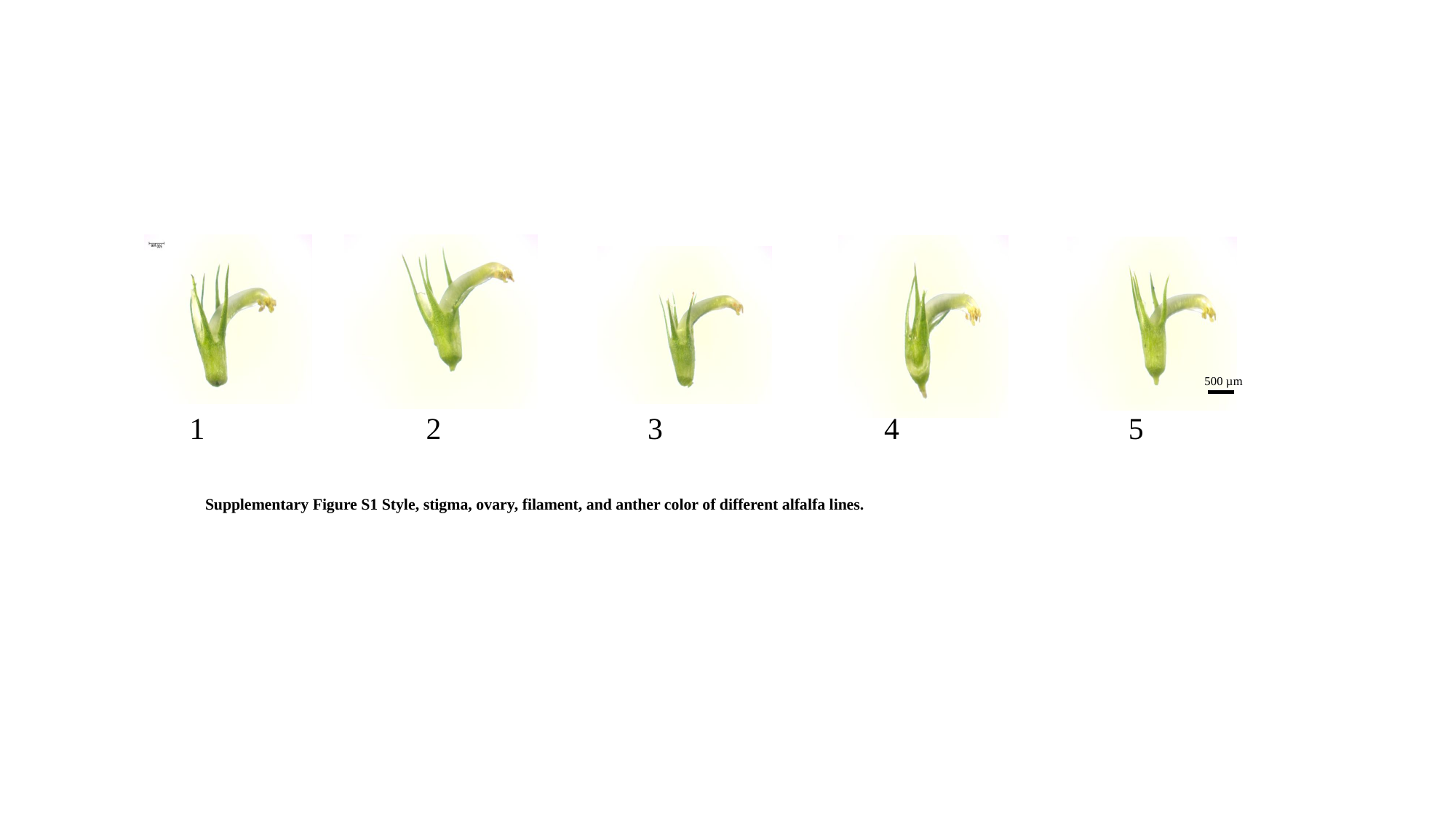

500 µm
1 2 3 4 5
Supplementary Figure S1 Style, stigma, ovary, filament, and anther color of different alfalfa lines.

## Slide 5
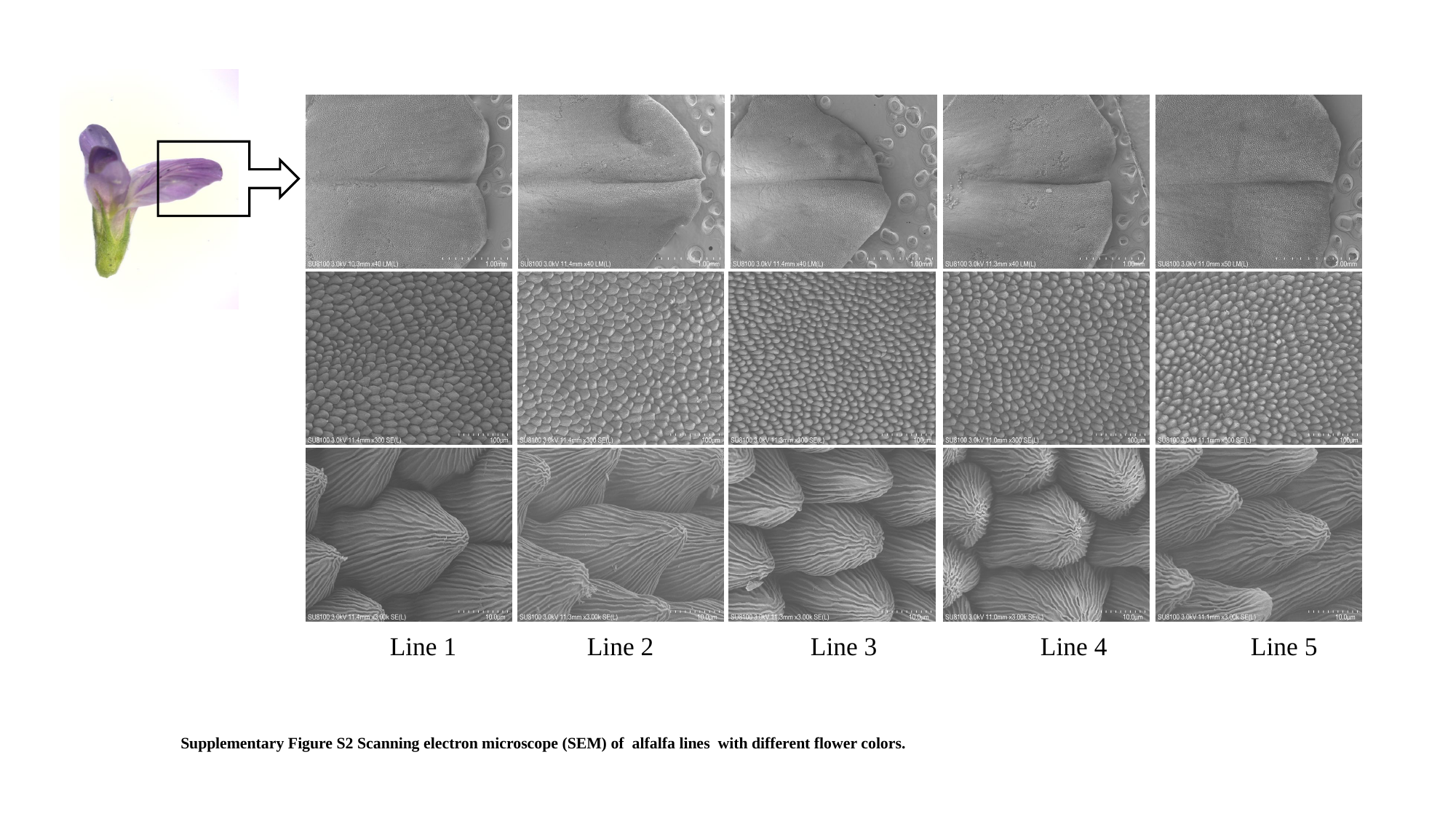

Line 1 Line 2 Line 3 Line 4 Line 5
Supplementary Figure S2 Scanning electron microscope (SEM) of alfalfa lines  with different flower colors.

## Slide 6
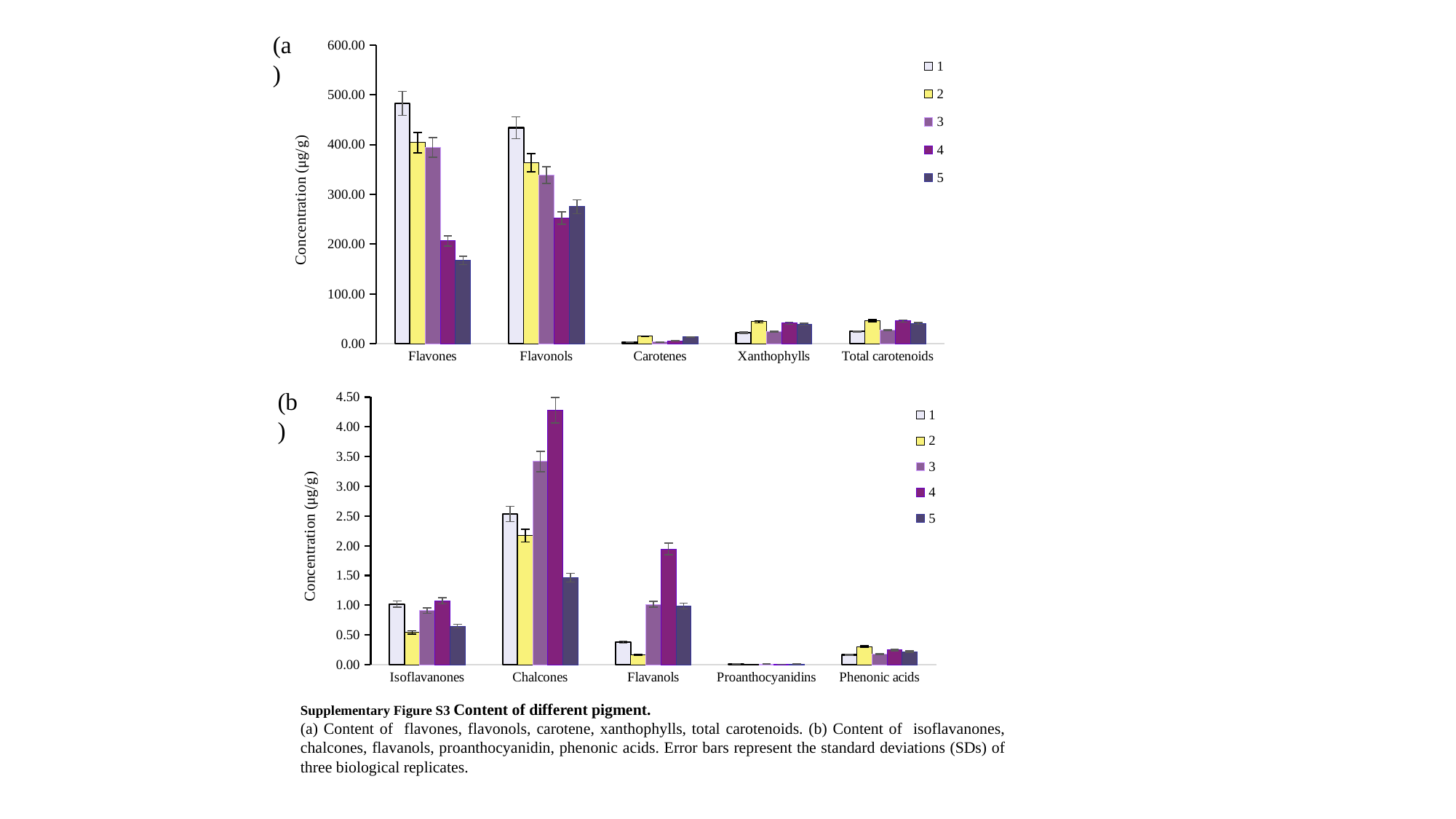

(a)
### Chart
| Category | 1 | 2 | 3 | 4 | 5 |
|---|---|---|---|---|---|
| Flavones | 482.82146280625335 | 404.080842704531 | 394.6212991818593 | 206.54807620579663 | 167.05225504477173 |
| Flavonols | 433.97882204652996 | 363.68411686332 | 339.0202179804564 | 252.62346015737 | 275.3883757926567 |
| Carotenes | 3.0860314446666663 | 14.813405687333331 | 3.5154612193333334 | 5.7174770399999995 | 13.414458267666665 |
| Xanthophylls | 22.02249873062767 | 44.12524048713601 | 24.035108971022 | 41.268308136787994 | 39.26135969139333 |
| Total carotenoids | 24.557564169794336 | 46.29544666470268 | 27.452063443725336 | 45.54772073401133 | 40.72499719142333 |(b)
### Chart
| Category | 1 | 2 | 3 | 4 | 5 |
|---|---|---|---|---|---|
| Isoflavanones | 1.0214647516807667 | 0.5426956848993 | 0.9085499848933999 | 1.0736981885958998 | 0.6491097237456667 |
| Chalcones | 2.5350654391666665 | 2.1702061775666666 | 3.416954472703333 | 4.279412597223334 | 1.46363750003 |
| Flavanols | 0.38209454402000004 | 0.16522894430666665 | 1.0168038464333333 | 1.9470059792333334 | 0.9873016806333332 |
| Proanthocyanidins | 0.016261111633333335 | 0.009563979466666667 | 0.012611511233333336 | 0.0074426857033333335 | 0.011176365933333335 |
| Phenonic acids | 0.166947767 | 0.303815782 | 0.18190865566666667 | 0.245897363 | 0.220003383 |Supplementary Figure S3 Content of different pigment.
(a) Content of flavones, flavonols, carotene, xanthophylls, total carotenoids. (b) Content of isoflavanones, chalcones, flavanols, proanthocyanidin, phenonic acids. Error bars represent the standard deviations (SDs) of three biological replicates.

## Slide 7
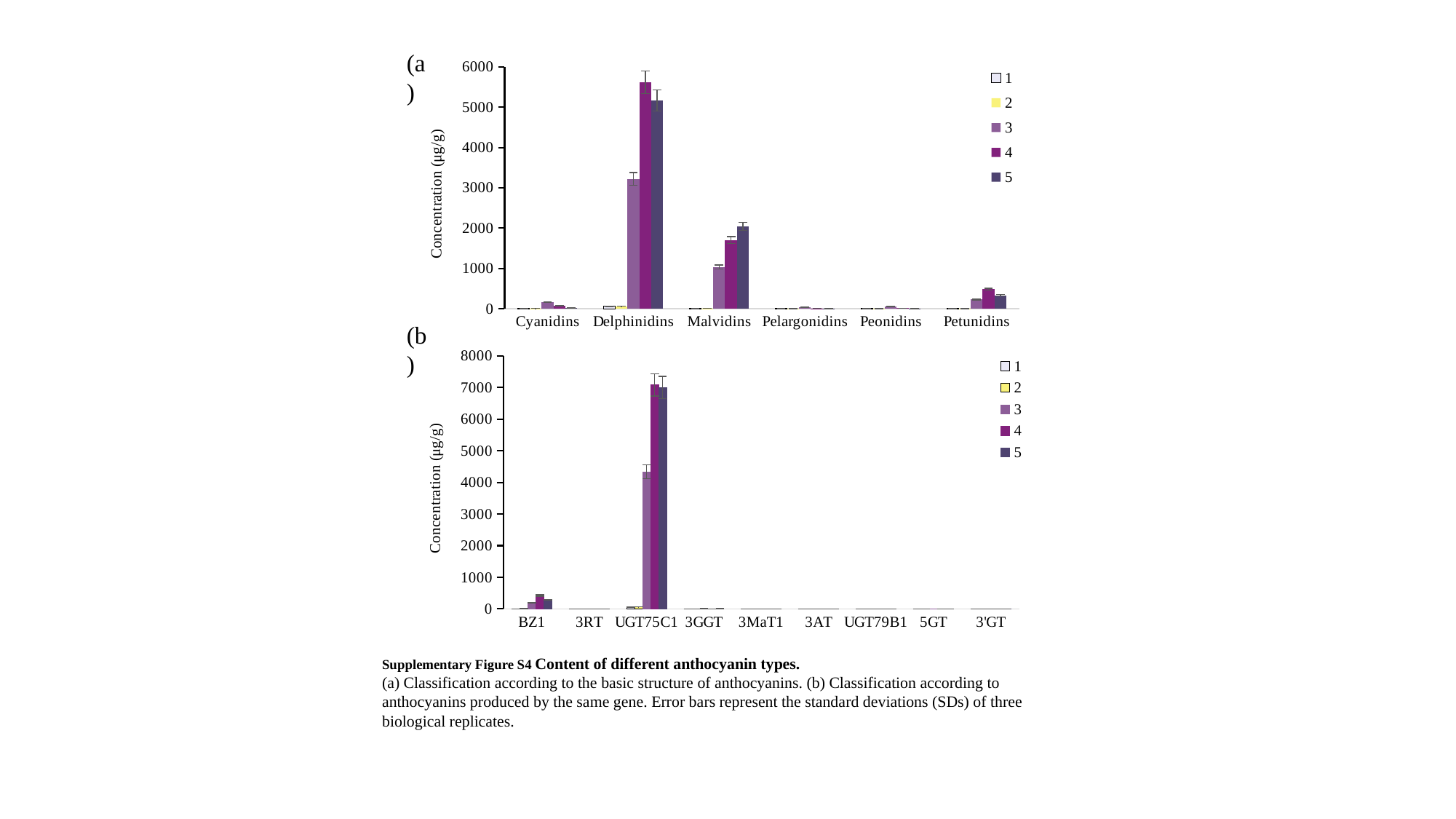

(a)
### Chart
| Category | 1 | 2 | 3 | 4 | 5 |
|---|---|---|---|---|---|
| Cyanidins | 3.7387840583800003 | 7.8270781842 | 159.34156705396 | 76.7543731724 | 23.28574013657 |
| Delphinidins | 55.622769571363335 | 62.24026371353 | 3220.173551171869 | 5618.69602013105 | 5166.942171033307 |
| Malvidins | 1.6794718127000001 | 5.721042966066666 | 1033.2121590095333 | 1703.0533985242 | 2041.9702095488665 |
| Pelargonidins | 0.2544458281 | 0.05238808811666667 | 38.30051648979 | 8.069272055436665 | 1.5753114447900003 |
| Peonidins | 0.09849811729666665 | 0.09281729200000001 | 52.978426034066665 | 10.176861948 | 3.847533761933333 |
| Petunidins | 2.9405899093000003 | 4.060191310433333 | 226.30808061733333 | 485.55061602316664 | 331.72291690813336 |(b)
### Chart
| Category | 1 | 2 | 3 | 4 | 5 |
|---|---|---|---|---|---|
| BZ1 | 2.4267557574133365 | 4.597642198906663 | 197.21830858866673 | 419.96317720666633 | 286.8530059681337 |
| 3RT | 0.0 | 0.0 | 0.1686253196666667 | 0.0 | 0.0 |
| UGT75C1 | 56.716762908400035 | 66.28336374380002 | 4331.073644166667 | 7072.714236613334 | 6998.201746380004 |
| 3GGT | 0.712183534283333 | 1.053287279533333 | 4.320887093599996 | 3.254093162766667 | 5.027850386266664 |
| 3MaT1 | 1.4247874008666634 | 0.9615228505666671 | 0.8335806427333337 | 2.2219803395666635 | 3.3047697675333367 |
| 3AT | 0.6681451037333334 | 0.6945667054100004 | 0.9775147058900002 | 0.3716324987699999 | 0.27269976159000003 |
| UGT79B1 | 0.20329518129999968 | 0.1587921786666667 | 0.6079574865999993 | 0.1351076687666667 | 0.32041996429999997 |
| 5GT | 0.00347663266333333 | 0.00348981643 | 0.000801289569 | 0.00418613521666667 | 0.00164839084 |
| 3'GT | 0.00683505454666667 | 0.00816072966666667 | 0.185470378333333 | 0.234804132 | 0.0211646660666667 |Supplementary Figure S4 Content of different anthocyanin types.
(a) Classification according to the basic structure of anthocyanins. (b) Classification according to anthocyanins produced by the same gene. Error bars represent the standard deviations (SDs) of three biological replicates.

## Slide 8
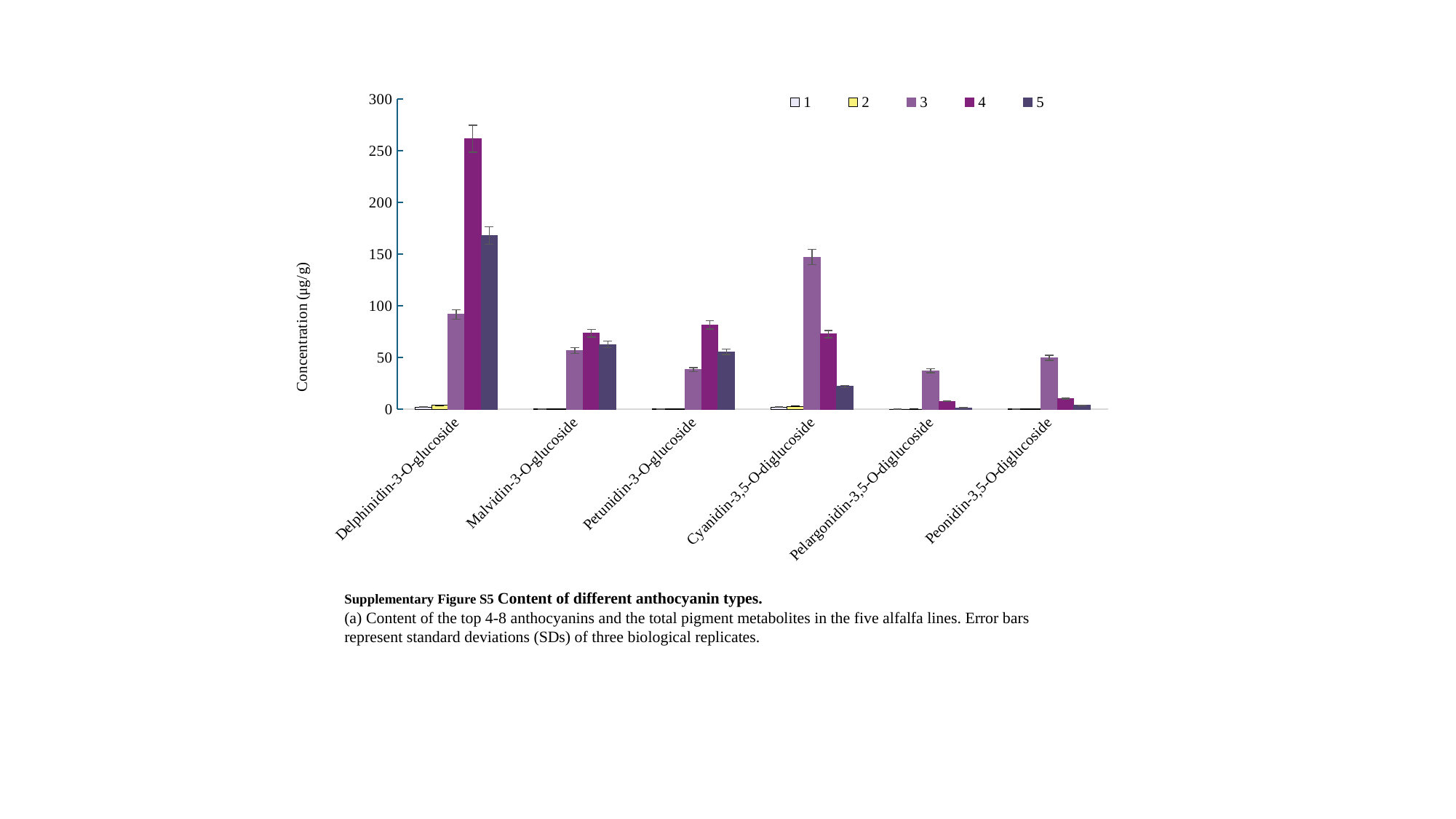

### Chart
| Category | 1 | 2 | 3 | 4 | 5 |
|---|---|---|---|---|---|
| Delphinidin-3-O-glucoside | 2.11361330666667 | 3.71753580333333 | 91.7466729666667 | 261.680024333333 | 168.076794666667 |
| Malvidin-3-O-glucoside | 0.12489246 | 0.289569500333333 | 56.8730673666667 | 73.5674298 | 62.7572388666667 |
| Petunidin-3-O-glucoside | 0.170787857666667 | 0.413995286666667 | 38.2696529666667 | 81.6058905 | 55.5355828333333 |
| Cyanidin-3,5-O-diglucoside | 1.95732551 | 2.75875465333333 | 147.251324 | 72.7265104333333 | 22.0021322666667 |
| Pelargonidin-3,5-O-diglucoside | 0.0561331483 | 0.0390102284666667 | 37.1991358666667 | 7.76444186333333 | 1.49178240666667 |
| Peonidin-3,5-O-diglucoside | 0.0866491367666667 | 0.092817292 | 49.8582106333333 | 9.99338431666667 | 3.77461504 |Supplementary Figure S5 Content of different anthocyanin types.
(a) Content of the top 4-8 anthocyanins and the total pigment metabolites in the five alfalfa lines. Error bars represent standard deviations (SDs) of three biological replicates.

## Slide 9
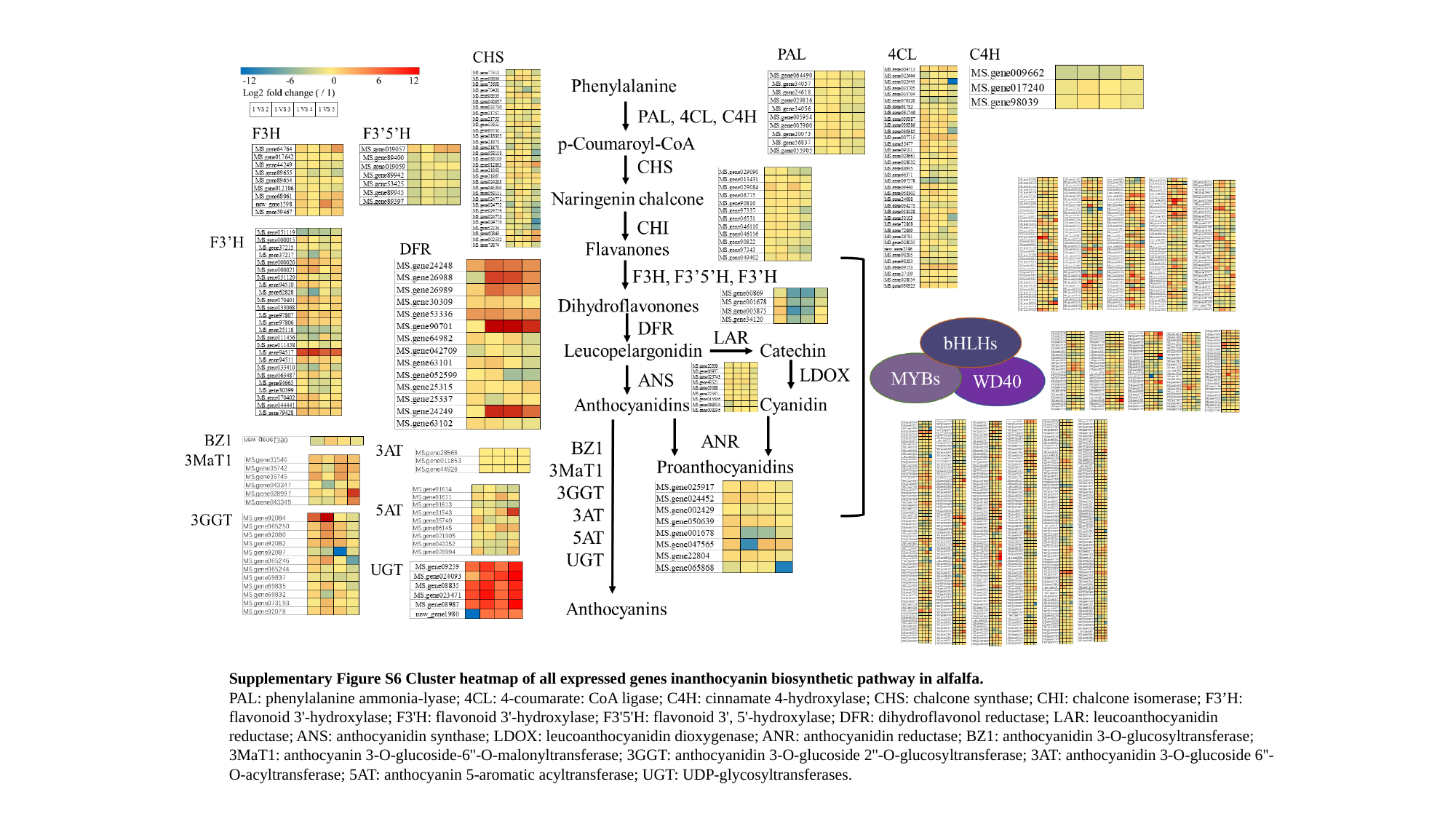

Supplementary Figure S6 Cluster heatmap of all expressed genes inanthocyanin biosynthetic pathway in alfalfa.
PAL: phenylalanine ammonia-lyase; 4CL: 4-coumarate: CoA ligase; C4H: cinnamate 4-hydroxylase; CHS: chalcone synthase; CHI: chalcone isomerase; F3’H: flavonoid 3'-hydroxylase; F3'H: flavonoid 3'-hydroxylase; F3'5'H: flavonoid 3', 5'-hydroxylase; DFR: dihydroflavonol reductase; LAR: leucoanthocyanidin reductase; ANS: anthocyanidin synthase; LDOX: leucoanthocyanidin dioxygenase; ANR: anthocyanidin reductase; BZ1: anthocyanidin 3-O-glucosyltransferase; 3MaT1: anthocyanin 3-O-glucoside-6''-O-malonyltransferase; 3GGT: anthocyanidin 3-O-glucoside 2''-O-glucosyltransferase; 3AT: anthocyanidin 3-O-glucoside 6''-O-acyltransferase; 5AT: anthocyanin 5-aromatic acyltransferase; UGT: UDP-glycosyltransferases.
